# Is the Empathy and Clarity Rating Scale a way to assess empathy in realistic clinical encounters?

**DOI:** 10.1080/10872981.2019.1680101

**Published:** 2019-10-14

**Authors:** Emily Kalms

**Affiliations:** Faculty of Medicine, Imperial College London, London, UK

**Keywords:** Communication skills, medical improvisation, objective structured clinical examination, empathy

Dear Editor,

The study by Terregino et al [1], outlines the formulation of the Empathy and Clarity Rating Scale (ECRS) and its piloted use to evaluate the impact of medical improv on communication skills. This scale assesses medical students on skills including gathering information and assisting patient decision-making. As a UK (UK) medical student and Associate Fellow of the Higher Education Academy, I have noticed that incorporating empathy into patient interactions is rarely assessed despite patients describing compassion as the most important quality sought from a physician [1].

The ECRS scale was tested with videos of students participating in objective structured clinical examinations (OSCEs) [1]. Comparisons between those with and without medical improv training suggested that theatre games improve medical communication by combining empathy and action so that patients feel compassion. Whilst medical improv isn’t widely available in UK medical schools, the underlying principles can be incorporated into simulation training. Healthcare professionals can improve students’ communication skills through simulation-based education [2] and students can be evaluated by the ECRS to assess the patient-centred approach.

The ECRS could also be used for a wide range of scenarios, including those appearing acutely medical. For example, a case about anaphylaxis allows the student to communicate with the patient and their family whilst prioritising acute management of the life-threatening condition. Additional notes could be added to the ECRS about how students balance emergency medical management with their communication towards the patient and family. This would allow students to act like physicians who manage medical problems alongside patient ideas, concerns and expectations. Focusing training on empathy ensures students display empathy in real-life scenarios as future doctors [3].

Additionally, a modified ECRS could be used to practice other realistic clinical encounters including consultations across a language barrier. Although interpreters should be available for consultations, issues including administrative errors mean this may not happen. Non-native speakers may not pick up on empathetic phrases and therefore rely on nonverbal, compassionate behaviours that cross-cultural boundaries [4].

To conclude, the ECRS appears to be a useful tool to assess medical student empathy in OSCEs to ensure students develop patient-centred communication skills [1]. Whilst medical improv is not commonly practiced in the UK, similar benefits could be gained from simulation training. The scale could be modified to practice scenarios including medical emergencies and consultations with communication barriers to ensure students feel and display empathy in realistic clinical encounters.
